# Microstructural Evolution of the NC-UHPC Near-Interface Composite Region Under Sequential Carbonation and Seawater Exposure

**DOI:** 10.3390/ma19163561

**Published:** 2026-08-21

**Authors:** Yan Zeng, Yubin Zheng, Zhu Wei, Foo Wei Lee, Sujie He, Yang Yang, Xiaoli Xie

**Affiliations:** 1 School of Civil Engineering, Guangxi Polytechnic of Construction, Nanning 530007, China; yanz1915@163.com (Y.Z.); he19962026@163.com (S.H.); sxwcyyy@163.com (Y.Y.);; 2 Department of Civil Engineering, Lee Kong Chian Faculty of Engineering and Science, Universiti Tunku Abdul Rahman, Kajang 43000, Malaysia; leefw@utar.edu.my; 3 School of Municipal Construction and Transportation, Guangxi Polytechnic of Construction, Nanning 530007, China; 18977980815@163.com; 4 Guangxi Yisheng Livable Building Materials Technology Co., Ltd., Nanning 530033, China

**Keywords:** ultra-high-performance concrete, pre-carbonation, seawater immersion, elemental redistribution, pore structure, microstructural evolution

## Abstract

**Highlights:**

- Carbonate signals intensified without dominant crystalline salt-attack products.
- Initial chemical heterogeneity controls preferential multi-ion transport.
- Durability assessment should consider transport-relevant defects alongside carbonate enrichment.

**Abstract:**

The long-term durability of repair systems combining normal concrete (NC) and ultra-high-performance concrete (UHPC) in marine environments depends on the response of the near-interface composite region to sequential carbonation and seawater exposure. However, the effects of seawater immersion following pre-carbonation remain insufficiently understood. This study compared an unexposed reference (REF), specimens carbonated for 28 d (C28), and specimens carbonated for 28 d and then immersed in simplified artificial seawater for 60 d (C28-SW60) using X-ray diffraction, thermogravimetry, backscattered electron imaging with energy-dispersive X-ray spectroscopy, and mercury intrusion porosimetry. Pre-carbonation promoted portlandite consumption, carbonate formation, and pore refinement. Subsequent seawater immersion further enhanced calcite-related diffraction and carbonate decomposition signals, while no typical crystalline salt-attack product was detected as dominant. The initial Ca-rich-to-Si-rich gradient from the NC side through the overlay transition zone to the UHPC side was accompanied by marked Cl accumulation and further S and Mg enrichment and redistribution. After seawater immersion, the measured total intrusion volume increased from 0.026 to 0.043 mL/g, the volume-based median pore-entry diameter increased from 27.49 to 58.42 nm, and the >1000 nm pore-volume fraction reached 39.82%, a change consistent with a shift toward coarser mercury-accessible pore entries. Together, the results link the initial heterogeneity of the NC–Overlay transition zone (OTZ)–UHPC region to a sequence-dependent response in which carbonate enrichment coexisted with multi-ion redistribution and transport-relevant defects, distinguishing carbonate accumulation from sustained near-interface refinement.

## 1. Introduction

Reinforced concrete structures in marine environments are subjected to the coupled effects of chloride ingress, sulfate attack, carbonation, wetting–drying cycles, and fluctuations in temperature and humidity. These exposures can promote reinforcement corrosion, cover cracking, and concrete degradation, ultimately compromising structural durability. For marine infrastructure, including ports, sea-crossing bridges, offshore platforms, and coastal facilities, repairing deteriorated concrete with highly durable materials is therefore essential to extend service life and reduce maintenance costs. Ultra-high-performance concrete (UHPC), characterized by a low water-to-binder ratio, dense microstructure, high strength, and low permeability, has emerged as a promising material for the repair and protection of normal concrete (NC) structures in marine environments [1,2,3,4]. However, the long-term performance of NC–UHPC composite repairs depends not only on the bulk properties of UHPC, but also on the integrity and durability of the interface between the NC substrate and the UHPC overlay [5,6,7,8].

The NC–UHPC interface constitutes a highly heterogeneous transition region. The NC side typically contains coarse aggregates, mature hydration products, and a relatively porous matrix, whereas the UHPC side is characterized by a higher powder content, a lower water-to-binder ratio, dispersed steel fibers, and a denser microstructure. These differences in elastic modulus, shrinkage behavior, pore structure, and hydration product assemblage make the interface a critical region for stress transfer, crack propagation, and the transport of aggressive species [6,7,8,9]. Accordingly, interfacial bond performance is governed by substrate strength, surface roughness and treatment method, interfacial moisture state, UHPC rheology and consolidation, and overall construction quality [10,11,12,13,14,15,16]. Pull-off and double-shear tests conducted by Zhang et al. [10] showed that substrate strength and interface roughness strongly influence the tensile and shear failure behavior of NC–UHPC interfaces. Yang et al. [11] further reported that appropriately designed grooves enhance mechanical interlocking, whereas Helsel et al. [14] and Du et al. [15] demonstrated that surface preparation, UHPC flow and consolidation, and interfacial voids directly affect the bond quality of the overlay–substrate system.

Previous research on NC–UHPC interfaces has focused primarily on initial bond behavior, surface treatment, and loading conditions. Al-Madani et al. [8] systematically evaluated the effects of surface roughness, curing conditions, and cyclic exposure on UHPC–NC interfacial bonding. Yu et al. [9] elucidated the multiscale debonding mechanisms of NC–UHPC interfaces under tensile and shear loading, whereas Zhang et al. [13] investigated interfacial shear failure through combined shear testing and morphological characterization. Collectively, these studies identified interfacial roughness, mechanical interlocking, and interfacial continuity as key determinants of the mechanical performance of NC–UHPC composite systems. More recent studies on interfaces between newly cast UHPC and existing NC, UHPC wet joints, and fresh-on-fresh interfaces have further shown that interfacial porosity, microcrack density, moisture state, and the intergrowth of hydration products govern bond strength and damage evolution [17,18,19,20]. Nevertheless, most previous studies have examined interfaces without prior environmental conditioning or have concentrated on construction-related variables. Consequently, the microstructural evolution of NC–UHPC repair interfaces under sequential pre-carbonation and seawater multi-ion exposure remains poorly understood.

Under realistic marine service conditions, existing NC substrates and their repair interfaces rarely undergo a single deterioration process. Instead, they may first experience CO_2_ ingress and carbonation, followed by prolonged exposure to seawater containing Cl^−^, SO_4_^2−^, Mg^2+^, and Na^+^ [21,22,23]. Carbonation consumes portlandite, promotes CaCO_3_ formation, and alters pore structure, alkalinity, aluminate-phase stability, and chloride-binding capacity [24,25,26,27]. Blackshaw et al. [26] showed that prior carbonation accelerates subsequent chloride ingress and promotes chloride redistribution near the carbonation front. Similarly, studies of carbonated concrete under simulated marine exposure have identified a secondary maximum in chloride concentration profiles that is absent in uncarbonated concrete and is associated with carbonate distribution, pore-structure evolution, and chloride redistribution [27,28]. Comparative studies under different marine exposure regimes further demonstrate that exposure conditions strongly govern ionic transport and accumulation in concrete [29]. Collectively, these findings indicate that prior carbonation alters the chemical and transport conditions governing subsequent chloride ingress and multi-ion seawater attack.

Seawater attack is governed not by chloride alone but by the coupled action of multiple ions. Cl^−^ may be incorporated into chloride-bearing phases or remain dissolved in the pore solution, whereas SO_4_^2−^ can drive sulfate-related phase transformations and Mg^2+^ can destabilize Ca-rich hydrates [30,31,32,33,34,35,36]. De Weerdt et al. [30] showed that exposure to saline solutions can induce the leaching of Ca-bearing phases and the precipitation of calcium carbonate or calcium sulfate and, under specific chemical conditions, promote the formation of brucite, magnesium silicate hydrate (M–S–H), or hydrotalcite-like phases. Zhao et al. [33], Zhang et al. [34], and Liu et al. [35] likewise demonstrated that the combined action of chloride, sulfate, and magnesium salts substantially alters phase assemblage, chloride-binding behavior, and microstructural stability. The NC–Overlay transition zone (OTZ)–UHPC region combines distinct phase assemblages, pore structures, and transport pathways, but the effect of prior carbonation on its subsequent multi-ion response remains unresolved. In particular, it is unclear whether carbonation-related pore refinement persists during seawater exposure and whether the ensuing evolution is governed by dominant crystalline salt-attack products or by spatial ion redistribution and defect restructuring.

To address this gap, this study examined the NC–UHPC near-interface composite region under three exposure states: an unexposed reference condition (REF), accelerated carbonation for 28 d (C28), and accelerated carbonation for 28 d followed by simplified artificial seawater immersion for 60 d (C28-SW60). X-ray diffraction (XRD) and thermogravimetry (TG) characterized the phase and thermal responses. Backscattered electron imaging coupled with energy-dispersive X-ray spectroscopy (BSE–EDS) resolved local morphology and elemental redistribution, whereas mercury intrusion porosimetry (MIP) characterized mercury-accessible pore entries. The analysis treated the NC side, OTZ, and UHPC side as a coupled, chemically heterogeneous region and followed its evolution across the exposure sequence rather than considering carbonation and seawater action separately. It examined how the initial Ca-rich-to-Si-rich gradient and preferential defects conditioned subsequent multi-ion transport and whether carbonation-related pore refinement persisted during seawater immersion. This sequence-based, cross-technique interpretation links initial near-interface heterogeneity to subsequent phase, elemental, and pore-entry changes and clarifies why carbonate enrichment does not necessarily preserve near-interface refinement.

## 2. Materials and Methods

### 2.1. Materials

The NC was used as the substrate, and the UHPC as the repair overlay. The NC was designed to meet strength class C40 and achieved a 28 d compressive strength of 43.7 MPa, whereas the UHPC achieved 145.2 MPa at the same age. Table 1 presents the mixture proportions of the NC substrate and UHPC overlay, and Table 2 lists the principal chemical compositions of the cementitious constituents [37]. For the NC, crushed limestone (Nanning Tongdasheng Concrete Co., Ltd., Nanning, China) with a maximum particle size of 25 mm and manufactured sand (Nanning Tongdasheng Concrete Co., Ltd., Nanning, China) were used as the coarse and fine aggregates, respectively. The NC binder comprised ordinary Portland cement (P·II52.5, Fusui Xinning Conch Cement Co., Ltd., Chongzuo, China), ground granulated blast-furnace slag (Guangxi Xintumao Technology Co., Ltd., Nanning, China), and limestone powder (Guangxi Xintumao Technology Co., Ltd., Nanning, China). For the UHPC, river sand (China Resources Zhizhu Technology (Nanning) Co., Ltd., Nanning, China)was used as the sole aggregate, and the binder comprised P·II 52.5 ordinary Portland cement, ground granulated blast-furnace slag, and silica fume (Guangxi Xintumao Technology Co., Ltd., Nanning, China). Straight copper-coated steel fibers (Hengshui Zhongxiang Engineering Materials Co., Ltd., Hengshui, China) were incorporated at a volume fraction of approximately 1.95%. Polycarboxylate-based high-range water-reducing admixtures (No. 1, Guangxi Xingdu Construction Technology Co., Ltd., Nanning, China; No. 2, Jiangsu Sobute New Materials Co., Ltd., Nanjing, China) were used to adjust the workability of both mixtures, and municipal tap water was used as the mixing water.

### 2.2. Specimen Preparation

**Figure 1:**
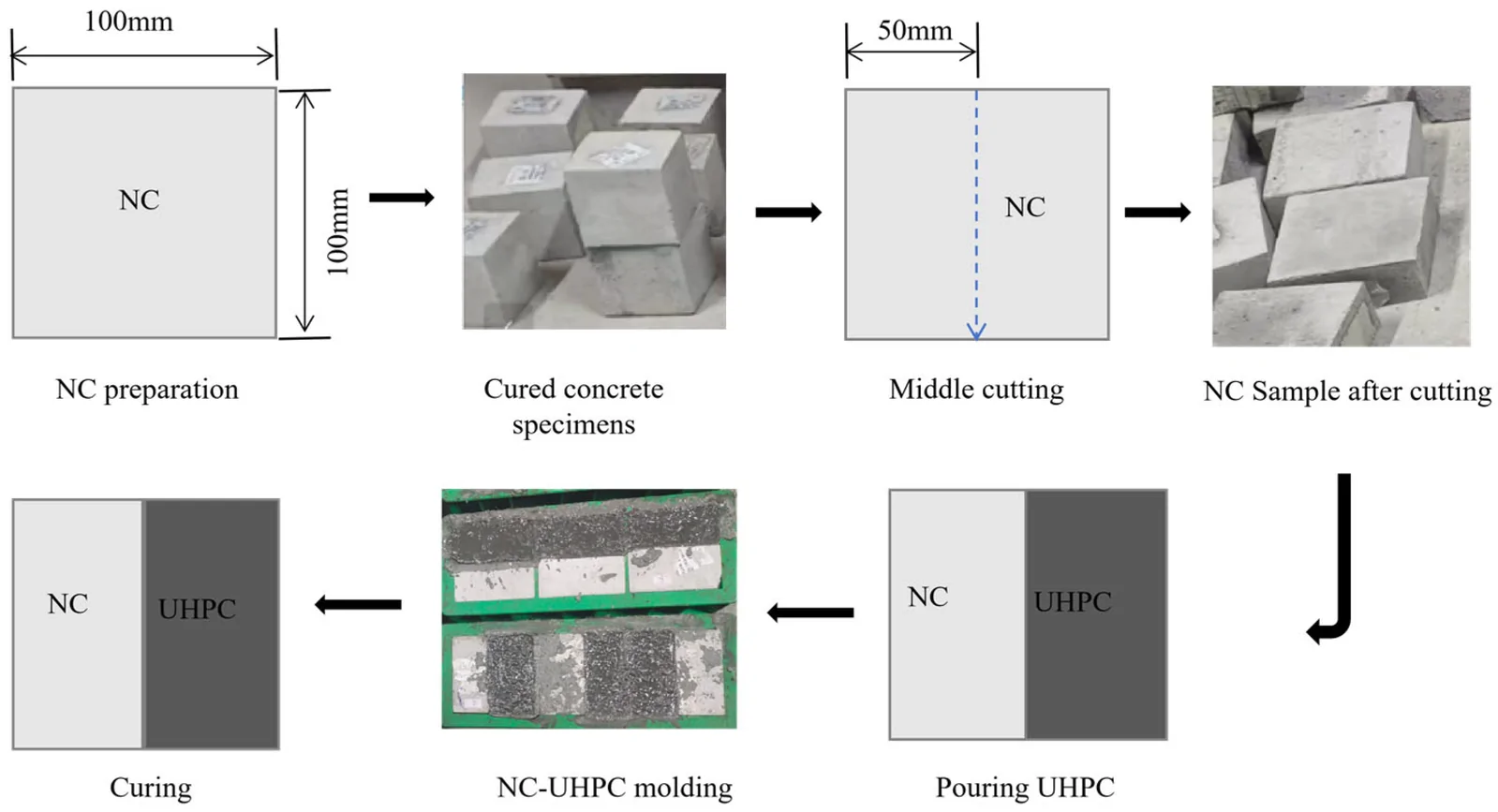
Preparation procedure for the NC-UHPC composite specimens.

Figure 1 illustrates the preparation procedure for the NC–UHPC composite specimens. The NC substrates were first cast in molds, compacted, and maintained for 24 h at 20 ± 5 °C and a relative humidity of at least 50%. The specimens were then demolded and cured under standard conditions of 20 ± 2 °C and a relative humidity of at least 95% until the NC reached an age of 28 d. Each NC specimen was subsequently cut along its mid-plane, and the resulting cut surface was used as the bonding surface for the UHPC overlay. The surface was rinsed with clean water to remove residual powder, debris, and loose particles. The prepared NC substrate was then repositioned in the mold, and the UHPC overlay was cast against the treated surface. The resulting composite specimens had final dimensions of 100 mm × 100 mm × 100 mm and comprised 50-mm-thick NC and UHPC layers, with the geometric NC–UHPC interface located at the specimen mid-plane. After UHPC casting, the composite specimens were maintained for 24 h, demolded, and cured under standard conditions until the UHPC reached an age of 28 d. The sequential casting and curing schedule represents the construction sequence of an NC–UHPC repair system, in which UHPC is placed on a previously cured NC substrate. After the subsequent 28 d curing period, the nominal ages of the NC and UHPC at the start of the environmental exposure program were 56 and 28 d, respectively. The specimens were subsequently subjected to the prescribed exposure regimes and sampled from the near-interface composite region for microstructural characterization.

### 2.3. Exposure Regimes

Three exposure regimes were established to investigate the microstructural evolution of the NC–UHPC near-interface composite region under sequential pre-carbonation and seawater immersion: an unexposed reference group (REF), accelerated carbonation for 28 d (C28), and accelerated carbonation for 28 d followed by simplified artificial seawater immersion for 60 d (C28-SW60). The REF group provided the initial microstructural baseline in the absence of external exposure, whereas the C28 group was used to isolate the effects of pre-carbonation on phase assemblage, thermal decomposition behavior, and pore structure. The C28-SW60 group was designed to reproduce, under accelerated conditions, a possible exposure sequence involving prior carbonation followed by multi-ion saline exposure in marine repair service. REF was characterized at the start of the exposure program, whereas C28 and C28-SW60 were characterized after an additional 28 and 88 d, respectively. The corresponding nominal NC/UHPC ages at characterization were 56/28 d for REF, 84/56 d for C28, and 144/116 d for C28-SW60. No unexposed specimens were retained to match the final chronological ages of C28 and C28-SW60; REF therefore serves as the initial unexposed baseline rather than an age-matched control.

After 28 d of curing following UHPC casting, the C28 and C28-SW60 specimens were exposed to accelerated carbonation for 28 d in a chamber maintained at a CO_2_ concentration of 20.0 ± 0.5%, a temperature of 20 ± 2 °C, and a relative humidity of 70 ± 5%. The chamber conditions and the 28 d test age are consistent with GB/T 50082 [38]. This exposure established a measurable pre-carbonated condition for examining how prior carbonation affected the subsequent response to artificial seawater. It was not intended to represent a specific duration of natural marine service because the relationship between accelerated and natural carbonation depends on the test protocol and material properties [39]. After carbonation, the C28 specimens were sampled for microstructural characterization, whereas the C28-SW60 specimens were immersed in simplified artificial seawater for 60 d. The reported Cl^−^ and SO_4_^2−^ concentrations in seawater from the Longmen Strait, Qinzhou Bay, are 10.86–10.90 and 1.44–1.74 g/L, respectively [40]. The artificial solution contained 108.8 g/L Cl^−^ and 15.9 g/L SO_4_^2−^, approximately 10 times the local concentrations. Industrial-grade NaCl and MgSO_4_·2H_2_O, each with a purity above 98%, were used to prepare the solution. This formulation was consistent with an accelerated protocol previously used for NC–UHPC composites [37]. The higher concentrations were selected to produce measurable multi-ion exposure within 60 d. They were not used to assign a field-equivalent exposure time because ion concentration can change transport rates, binding equilibria, and reaction kinetics. The results therefore describe the sequence and coupling of microstructural processes under intensified exposure rather than the rate of deterioration in natural seawater. The solution contained no added Ca^2+^, K^+^, HCO_3_^−^, or CO_3_^2−^. It therefore provided a controlled, intensified Cl^−^–SO_4_^2−^–Mg^2+^ exposure rather than reproducing the full ionic composition and carbonate chemistry of natural seawater. The solution was replaced every 15 d to limit changes in solution chemistry and maintain approximately stable ion concentrations. All immersion tests were conducted at room temperature. The characterization endpoints were defined relative to the initial 28 d UHPC curing period. REF was characterized at the end of this curing period without external exposure. C28 was characterized after a further 28 d of accelerated carbonation, whereas C28-SW60 was characterized after the same carbonation stage followed by 60 d of simplified artificial seawater immersion.

### 2.4. Characterization Methods

All characterization was performed as destructive endpoint measurements after each group reached its designated condition. The same physical specimen was therefore not measured both before and after exposure. For a given exposure condition, the specimens were subjected to the same prescribed environmental regime and exposure duration, although the specimen geometry and sampled volume differed among the characterization routes. Within each technique, the specimen form, nominal sampling location, and preparation procedure were kept consistent across REF, C28, and C28-SW60. The exposure-related differences were therefore interpreted within each technique, whereas the cross-technique results were integrated qualitatively at the exposure-condition level. For each condition, one powder aliquot was analyzed by XRD and one by TG–DTG, while BSE–EDS and MIP were each performed on one separate 10 mm × 10 mm× 10 mm near-interface cube. The quantitative results are therefore reported as individual endpoint measurements without error bars for specimen-to-specimen variability or tests of statistical significance.

#### 2.4.1. X-Ray Diffraction

**Figure 2:**
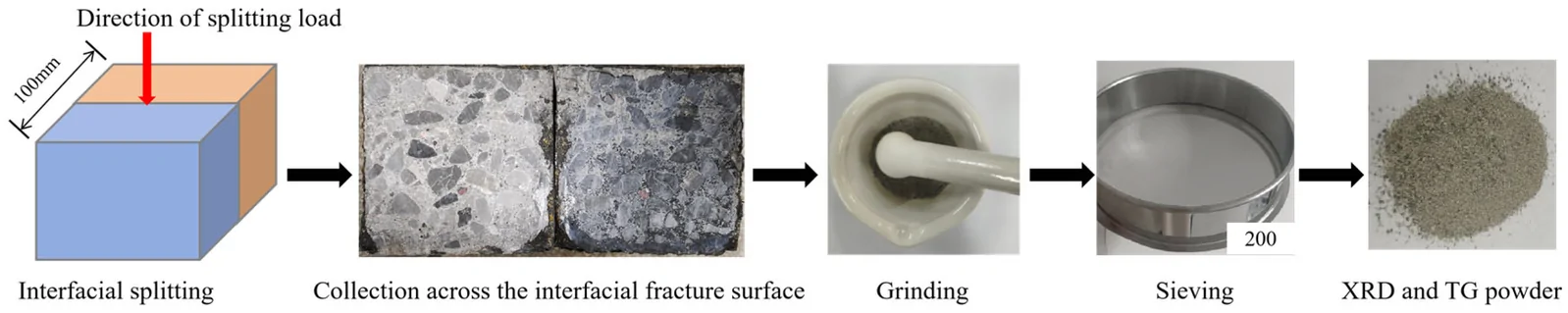
Preparation procedure for the XRD and TG powder samples.

The XRD was used to characterize the crystalline phase assemblage and its evolution in the NC–UHPC near-interface composite region. After completion of the prescribed exposure regime, the composite specimens were subjected to splitting until failure occurred along the NC–UHPC interface. After the specimens were split along the NC–UHPC interface, each newly exposed fracture surface was placed above a clean collection sheet. A clean, flat-edged sampling tool was used under dry conditions to remove material systematically across the full 100 mm × 100 mm projected interfacial area. The operator traversed the fracture surface in consecutive overlapping passes to cover the entire interface without preferentially selecting paste-rich or aggregate-rich locations. Collection was restricted to material directly attached to the newly exposed fracture surface, without deeper localized excavation into either the NC or the UHPC. Because the irregular fracture surface was sampled manually rather than with a depth-controlled grinder, the collected material could not be assigned a uniform geometric thickness. The powder therefore represents an area-averaged fracture-surface sample of the NC–UHPC near-interface composite region rather than a depth-resolved layer. All collected powder and fragments were combined and ground using a porcelain mortar and pestle. The material was passed through a 200-mesh sieve, and the retained fraction was reground and resieved until it passed through the sieve. The resulting powder was thoroughly mixed before separate aliquots were taken for XRD and TG–DTG analysis, as illustrated in Figure 2. XRD patterns were collected using a Rigaku MiniFlex 600C diffractometer (Rigaku Electric Co., Ltd., Tokyo, Japan) with Cu Kα radiation (λ = 1.5406 Å). The measurements were conducted over a 2θ range of 10–80° at a scanning rate of 5°/min.

#### 2.4.2. Thermogravimetry

The TG was used to characterize the thermal decomposition behavior of hydration products, carbonation products, and potential products associated with salt attack in the NC–UHPC near-interface composite region. Separate aliquots of the same homogenized interfacial powder were used for XRD and TG–DTG, as illustrated in Figure 2. TG measurements were performed using a TGA Q5000 thermogravimetric analyzer (TA Instruments, New Castle, DE, USA) equipped with a Platinum-HT pan under a flowing N_2_ atmosphere. The N_2_ flow rates for the balance and sample gases were 40.0 and 50.0 mL/min, respectively. The samples were heated from 30 to 820 °C at a rate of 20 °C/min. The TG curves were normalized to the initial sample mass and expressed as mass percentages, whereas the derivative thermogravimetry (DTG) curves were used to identify the principal mass-loss events within different temperature ranges.

#### 2.4.3. Backscattered Electron Imaging Coupled with Energy-Dispersive X-Ray Spectroscopy

**Figure 3:**
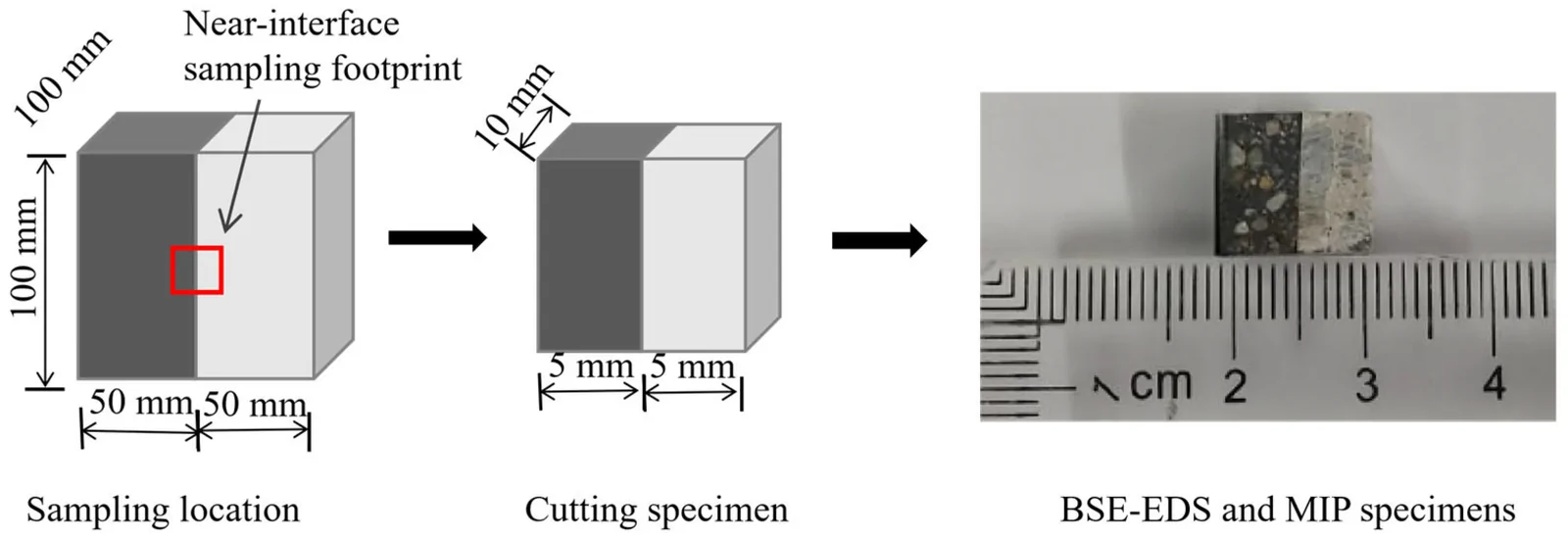
Sampling locations and specimens for BSE-EDS and MIP.

BSE–EDS was used to characterize the morphology and elemental distribution of the NC–UHPC near-interface composite region. After 28 d of curing following UHPC casting, the composite specimens were cut into 10 mm × 10 mm × 10 mm cubes centered on the geometric interface, with each cube containing the NC side, the OTZ, and the UHPC side (Figure 3). The cubes were subjected to the exposure regimes described in Section 2.3. After seawater immersion, excess surface solution was gently removed using a lint-free cloth that had been moistened with the same artificial seawater and squeezed. The C28-SW60 specimens were not rinsed with water to limit the removal and redistribution of soluble ions during post-exposure handling. The observation surface spanning the NC, OTZ, and UHPC was ground sequentially with silicon carbide abrasive papers of progressively finer grit and polished with an alumina suspension. Residual polishing material was removed with anhydrous ethanol. The specimens were then dried at 60 ± 5 °C for at least 24 h and sputter-coated with gold. The same observation-plane orientation and surface preparation procedure were applied to REF, C28, and C28-SW60. BSE images and EDS elemental maps were acquired using a ZEISS Sigma 360 scanning electron microscope (Zeiss, Oberkochen, Germany) equipped with an Oxford Xplore 30 detector (Oxford Instruments, Oxford, UK) at an accelerating voltage of 15 kV. The mapped areas spanned the NC side, the OTZ, and the UHPC side. Because the study compared the images qualitatively, crack width, crack density, and crack connectivity were not quantified.

#### 2.4.4. Mercury Intrusion Porosimetry

MIP was used to characterize the connected, mercury-accessible pore network and pore-entry size distribution of the NC–UHPC near-interface composite region. Separate 10 × 10 × 10 mm cubes were used for MIP and BSE–EDS. The MIP cubes had the same nominal near-interface location and initial dimensions as the specimens prepared for BSE–EDS, with each cube containing the NC side, the OTZ, and the UHPC side, as illustrated in Figure 3. Measurements were performed using an AutoPore IV 9500 mercury porosimeter (Micromeritics Instrument Corporation, Norcross, GA, USA) at a maximum intrusion pressure of 228 MPa, providing a nominal pore-entry diameter range of 5–8 × 10^5^ nm. Before testing, the specimens were oven-dried at 105 ± 1 °C for 24 h to remove free water from the accessible pore network. Because each cube contained the NC side, the OTZ, and the UHPC side, the reported parameters represent the mercury-accessible response of the fixed near-interface composite volume rather than the pore structure of any constituent region in isolation. All exposure groups underwent the same drying and intrusion procedure. Nevertheless, drying-induced alteration and ink-bottle effects cannot be excluded and were considered when interpreting the differences among the exposure conditions.

## 3. Results

### 3.1. Phase Assemblage and Thermal Decomposition

#### 3.1.1. X-Ray Diffraction Analysis

**Figure 4:**
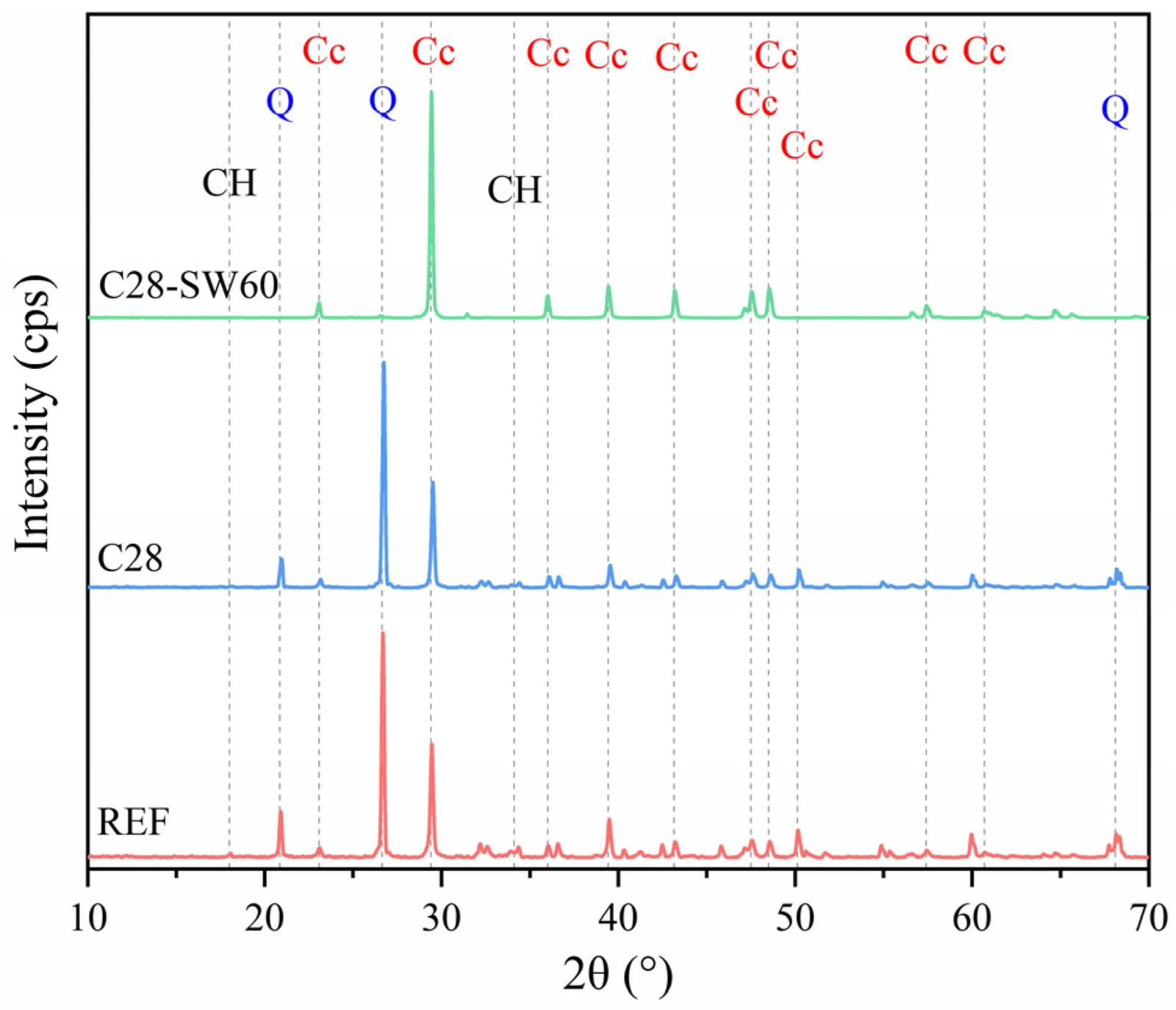
XRD patterns of the NC-UHPC near-interface region under different exposure regimes. Q = quartz, Cc = calcite, CH = portlandite.

Figure 4 presents the XRD patterns of the NC–UHPC near-interface composite region for the REF, C28, and C28-SW60 groups. The crystalline phase assemblage of REF was dominated by quartz and calcite, with a small amount of portlandite (CH). The intense reflection at approximately 26.6° was assigned to quartz and originated mainly from siliceous aggregates and other crystalline SiO_2_ constituents in the NC and UHPC. The reflections at approximately 29.4°, 39.4°, 43.1°, 47.5°, and 48.5° were assigned to calcite, whereas the weak reflections at approximately 18.0° and 34.1° corresponded to CH. Because the NC mixture contained both limestone powder and limestone coarse aggregate, the calcite detected in REF primarily represented the inherent carbonate background of the composite rather than a product of environmental carbonation. Accordingly, the normalized peak areas describe relative diffraction contributions within the composite powders rather than absolute phase contents. Previous studies have similarly reported pronounced initial calcite signals in uncarbonated cementitious materials containing limestone powder or other carbonate-bearing constituents [41,42].

After 28 d of accelerated carbonation, the phase assemblage of C28 remained dominated by quartz and calcite, with minor residual CH. No additional major crystalline phase was detected within the resolution of the XRD analysis. The principal changes were the attenuation of the CH reflections and the intensification of the main calcite reflection near 29.4°. The normalized integrated area of the calcite peak window increased from 13.21% for REF to 15.70% for C28, indicating a greater relative diffraction contribution from crystalline calcite after pre-carbonation. This trend is consistent with the consumption of CH and the formation of CaCO_3_ during the carbonation of cementitious materials [38,39,40]. In addition to the direct carbonation of CH, other Ca-bearing hydrates, including calcium (alumino)silicate hydrate (C–(A)–S–H), may have undergone decalcification, producing Si-rich phases and poorly crystalline or amorphous carbonates that could not be resolved as distinct XRD reflections [43,44].

The calcite diffraction signal strengthened further in C28-SW60. In addition to the marked intensification of the main reflection near 29.4°, other calcite reflections at approximately 36.0°, 39.4°, 43.2°, 47.5°, 48.5°, and 57–60° became more pronounced. The normalized integrated area of the 29.4° calcite peak window increased from 15.70% for C28 to 42.24% for C28-SW60, indicating a stronger relative diffraction contribution from crystalline calcite under the C28-SW60 condition. Previous studies have shown that Mg^2+^, Cl^−^, and SO_4_^2−^ can alter the dissolution, transport, and reprecipitation of Ca-bearing phases and influence CaCO_3_ crystallization and polymorphism [45,46]. The stronger calcite signal is therefore consistent with the redistribution and further crystallization of carbonates present after pre-carbonation, together with the dissolution and reprecipitation of Ca-bearing constituents during subsequent multi-ion exposure. The CH reflections near 18.0° and 34.1° weakened further in C28-SW60, with the latter approaching the background level. This result indicates a further depletion of detectable crystalline portlandite under sequential pre-carbonation and seawater immersion. In addition to the carbonation of CH during the initial exposure stage, seawater immersion may have promoted the consumption of residual CH through Ca^2+^ leaching and secondary reactions involving Mg^2+^ and SO_4_^2−^ [46,47]. Studies of seawater-exposed UHPC have similarly shown that continued hydration, ion transport, and localized secondary-product precipitation can occur concurrently, resulting in time-dependent and spatially heterogeneous phase evolution [48].

Minor reflections and local changes in peak shape were also observed near 23° and within the range of 31–32° for C28-SW60. However, secondary calcite reflections, residual unhydrated clinker minerals, and low-abundance salt-binding phases may overlap within these ranges, precluding unambiguous phase identification from the present XRD patterns alone. Although seawater-derived Cl^−^ and SO_4_^2−^ can promote the formation of Friedel’s salt and ettringite, respectively, while gypsum, brucite, and M–S–H may develop under prolonged exposure or more severe magnesium salt attack [46,47,49], no distinct reflections could be unequivocally assigned to these products. These phases were therefore not detected as dominant crystalline products in the NC–UHPC near-interface composite region after 60 d of seawater immersion. The XRD results were subsequently assessed together with the TG–DTG and BSE–EDS results to clarify the relationships among carbonate enrichment, multi-ion ingress, and potential salt-related reactions.

#### 3.1.2. Thermogravimetry Analysis

**Figure 5:**
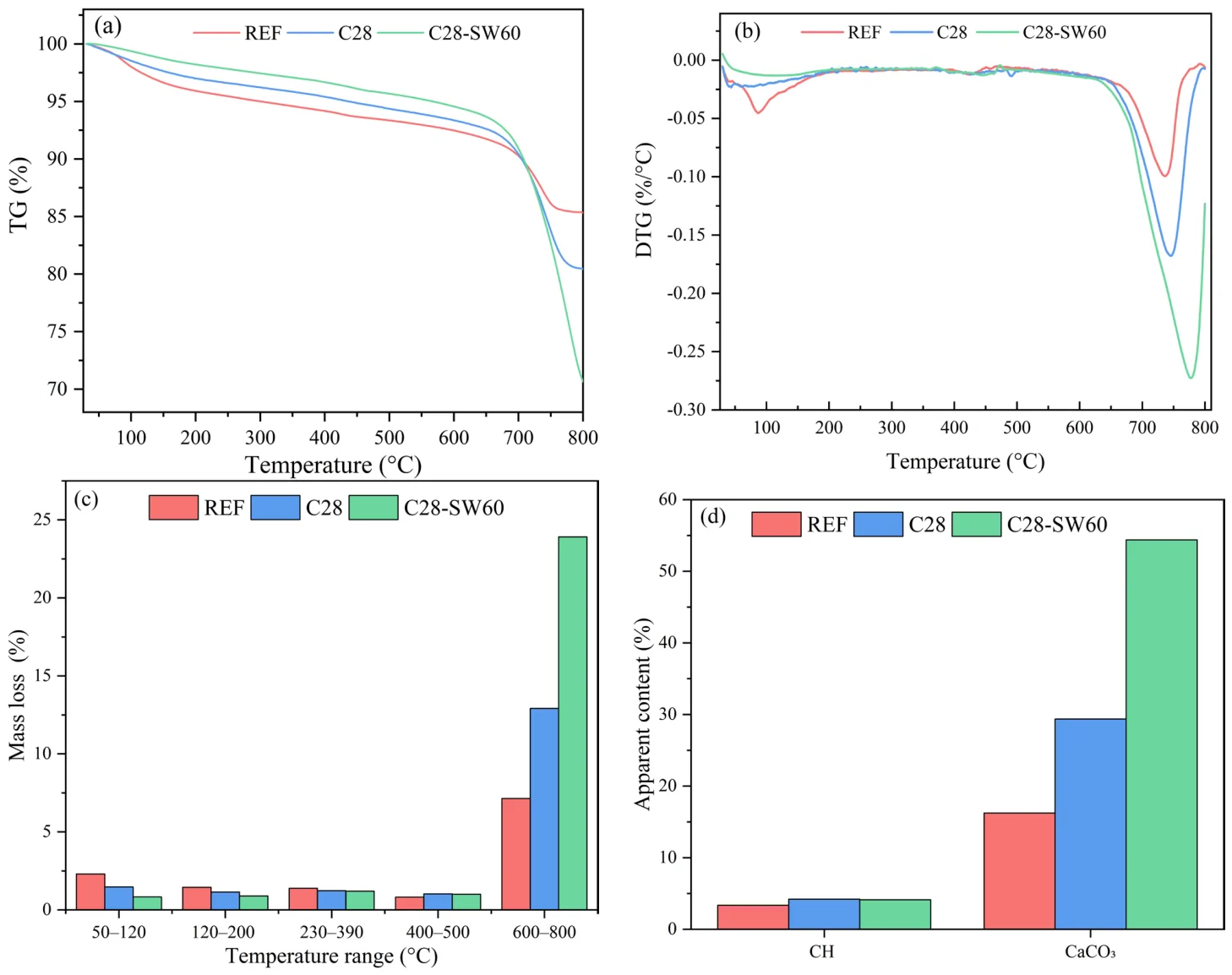
TG-DTG results of the NC-UHPC near-interface composite region: (**a**) TG curves; (**b**) DTG curves; (**c**) mass loss of typical temperature ranges; and (**d**) apparent CH and CaCO_3_ contents.

Figure 5 presents the TG–DTG results for the NC–UHPC near-interface composite region under the three exposure regimes. As shown in Figure 5a, all groups exhibited continuous mass loss with increasing temperature, although the magnitude varied substantially. The total mass losses over 30–800 °C were 14.66%, 19.55%, and 29.35% for REF, C28, and C28-SW60, respectively. The higher mass loss of C28 relative to REF indicates that pre-carbonation altered the relative abundance and composition of thermally decomposable constituents within the near-interface region. The further increase in C28-SW60 suggests additional transformation and/or accumulation of thermally decomposable products during seawater immersion. These changes may involve continued hydration of residual unhydrated constituents in the UHPC, ion-induced transformation of hydration products, and further evolution of carbonate-bearing phases [45,47,50].

Figure 5b shows broad high-temperature DTG minima above approximately 700 °C, and the largest differences among the three conditions occurred within 600–800 °C. The peak position and the concurrent calcite reflections detected by XRD support assignment of this interval as carbonate-decomposition-dominated. Its principal mass loss was therefore attributed to the decarbonation of carbonate-bearing constituents rather than to dehydroxylation [42,45]. REF already exhibited a substantial mass-loss event within this range because of the initial CaCO_3_ background introduced by the limestone powder and limestone coarse aggregate in the NC. Calcite originating from limestone-bearing raw materials can produce a pronounced high-temperature decomposition signal even in uncarbonated cementitious materials. Therefore, the carbonate-related mass loss measured by TG cannot be attributed solely to exposure-induced carbonation [42]. After 28 d of accelerated carbonation, the mass loss of C28 within this range increased markedly, consistent with the carbonation of CH and other Ca-bearing hydrates, including C–(A)–S–H, and the associated formation of CaCO_3_ [44,51]. The further increase in C28-SW60 agrees with the enhanced calcite diffraction signal observed by XRD and indicates a greater relative thermal contribution from carbonate-bearing constituents after seawater immersion. Seawater exposure can promote continued hydration of residual unhydrated phases and alter hydrate assemblage and dissolution–reprecipitation processes through the action of Cl^−^, SO_4_^2−^, and Mg^2+^ [47,50]. In pre-carbonated calcium silicate systems, subsequent seawater exposure may also influence CaCO_3_ precipitation, polymorphic composition, and crystallization behavior [45]. The enhanced carbonate decomposition signal in C28-SW60 may therefore reflect the combined effects of further crystallization of pre-existing carbonates, migration and reprecipitation of Ca-bearing constituents, and changes in carbonate polymorphism.

For a more detailed comparison, Figure 5c presents the mass losses within five representative temperature ranges. Within 50–120 °C, the mass losses of REF, C28, and C28-SW60 were 2.30%, 1.47%, and 0.83%, respectively. This range mainly captures the release of evaporable, adsorbed, and weakly bound water, together with partial low-temperature dehydration of C–(A)–S–H, ettringite-type (AFt), and monosulfate-type (AFm) phases. Because these thermal events strongly overlap below approximately 200 °C, their individual contributions cannot be quantitatively assigned to specific phases [42,52]. The progressive decrease in mass loss therefore indicates a lower combined contribution from low-temperature water-release events. This trend may reflect carbonation-induced transformation of hydrous phases, decalcification and restructuring of C–(A)–S–H, and progressive carbonate enrichment [42,51].

Within 120–200 °C, the mass losses were 1.45%, 1.15%, and 0.89% for REF, C28, and C28-SW60, respectively. This range contains overlapping contributions from gypsum, AFt/AFm-type aluminate hydrates, and other low-temperature dehydration processes [52]. Seawater-derived Cl^−^ and SO_4_^2−^ can alter AFm-phase composition and promote Friedel’s salt formation or AFt stabilization [47]. However, C28-SW60 exhibited no increase in mass loss within this range, providing no evidence of a pronounced thermal response associated with substantial net accumulation of gypsum or aluminate-bearing salt-reaction products under the present exposure conditions. Similarly, the mass loss within 230–390 °C decreased from 1.39% for REF to 1.23% for C28 and remained nearly unchanged at 1.20% for C28-SW60. This range may include contributions from the progressive dehydration and dehydroxylation of chloride-bearing AFm (Cl-AFm) phases, including Friedel’s salt, as well as partial dehydroxylation of brucite or other Mg-bearing hydrates [53]. The absence of a new or markedly intensified intermediate-temperature mass-loss feature in C28-SW60, together with the lack of distinct XRD reflections attributable to Friedel’s salt or brucite, provides no clear evidence for their extensive accumulation.

The 400–500 °C interval is primarily associated with portlandite dehydroxylation. The mass losses of REF, C28, and C28-SW60 were 0.82%, 1.02%, and 1.00%, respectively. Conversion based on the stoichiometric release of H_2_O yielded apparent CH-equivalent contents of 3.37%, 4.21%, and 4.12%, respectively (Figure 5d). These values contrast with the progressive weakening of the CH reflections measured by XRD. In seawater-containing cementitious systems, portlandite dehydroxylation has been assigned to approximately 410–510 °C, while an Mg(OH)_2_-related feature near 400 °C and overlapping carbonate- and M–S–H-related mass loss extending toward 600 °C have also been reported [54]. Amorphous or poorly crystalline CaCO_3_ and metastable carbonate polymorphs may likewise decompose within approximately 400–600 °C in carbonated cementitious materials [42,45]. These overlapping events, together with baseline selection and local compositional variability, affect fixed-range integration. The calculated values are therefore reported as apparent CH-equivalent contents rather than absolute portlandite contents.

By contrast, the carbonate-decomposition-dominated range of 600–800 °C exhibited the largest differences among the groups. The corresponding mass losses were 7.14%, 12.91%, and 23.92% for REF, C28, and C28-SW60, respectively. Conversion based on the stoichiometric release of CO_2_ during CaCO_3_ decomposition yielded apparent CaCO_3_-equivalent contents of 16.24%, 29.37%, and 54.39%, respectively (Figure 5d). The measured values show that the high-temperature carbonate decomposition signal was higher after pre-carbonation and increased further after subsequent immersion. This trend agrees with the stronger calcite reflections in C28-SW60 and indicates a greater relative contribution from carbonate-bearing constituents. Carbonate redistribution, reprecipitation, and further crystallization may all have contributed to this response. Because the exposure solution contained no added Ca^2+^, HCO_3_^−^, or CO_3_^2−^, these changes should not be interpreted as calcite precipitation under the complete carbonate chemistry of natural seawater. The measurements do not distinguish the relative contributions of these pathways or determine how the omitted seawater constituents would alter the magnitude, kinetics, or polymorphic distribution of the response. Overall, the TG–DTG and XRD results identify stronger carbonate-related signals as the principal phase response of the NC–UHPC near-interface composite region under the applied sequential exposure. In contrast, the low- and intermediate-temperature ranges provided no evidence that typical crystalline products associated with salt attack became the dominant thermally decomposable constituents.

### 3.2. Morphology and Elemental Redistribution

#### 3.2.1. BSE Morphology

**Figure 6:**
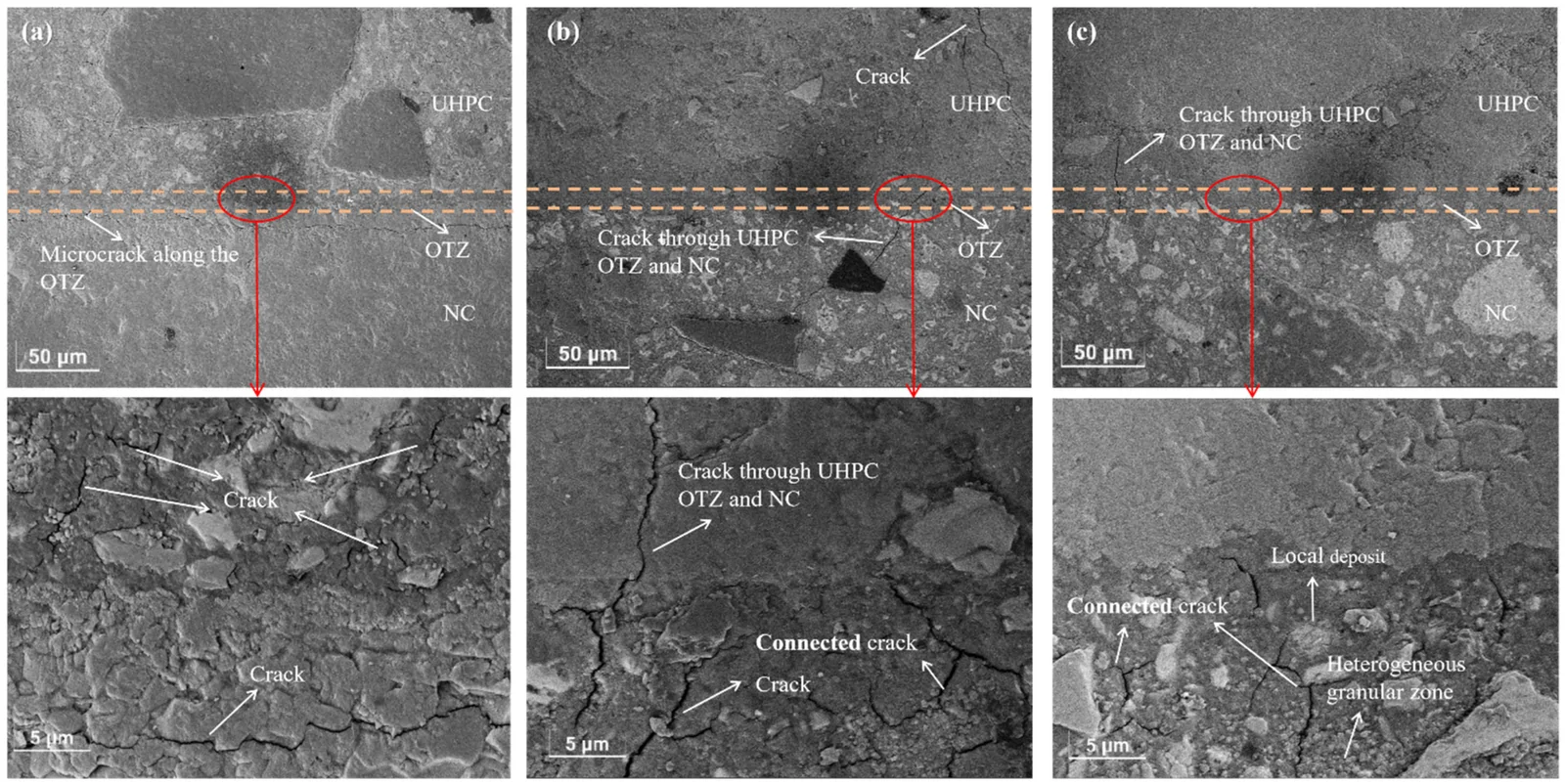
BSE micrographs of the NC–UHPC near-interface composite region: (**a**) REF, (**b**) C28, and (**c**) C28-SW60.

Figure 6 presents BSE micrographs of the NC–UHPC near-interface composite region for the REF, C28, and C28-SW60. Across the three exposure conditions, the micrographs differed qualitatively in crack-like features, matrix continuity, and localized deposit-like features. The near-interface region of REF (Figure 6a) was generally continuous, without extensive connected cracks or clear interfacial separation. Isolated fine cracks and local discontinuities were observed along some particle boundaries, likely reflecting the inherent contrasts in constituent properties, aggregate distribution, and interface formation within the NC–UHPC composite. Overall, REF retained a relatively intact baseline morphology.

After 28 d of accelerated carbonation, C28 (Figure 6b) exhibited more pronounced crack-like defects and greater spatial variation in gray level. Several defects followed particle boundaries or locally discontinuous matrix regions, and cracking was visible around some particle edges. Relative to REF, the crack-like features appeared more numerous and locally interconnected. These observations indicate that any carbonate precipitation and localized pore filling induced by pre-carbonation did not produce uniform densification throughout the near-interface region. Consistent with the XRD and TG–DTG results, the enhanced carbonate-related signals in C28 are therefore more appropriately interpreted as evidence of spatially heterogeneous phase transformation and localized structural modification rather than uniform interfacial densification.

C28-SW60 (Figure 6c) showed greater morphological heterogeneity than C28. Granular and deposit-like features occurred near cracks, particle boundaries, and discontinuous matrix regions, but they did not form a continuous product layer. Higher-magnification images showed granular accumulations, slit-like defects, and locally connected crack-like features. Thus, localized precipitation coexisted with crack-like discontinuities rather than uniformly filling the near-interface region. Under C28-SW60, carbonate enrichment, localized deposition, and local crack-like defects therefore coexisted without forming a continuous crystalline salt-attack layer.

#### 3.2.2. EDS Mapping

**Figure 7:**
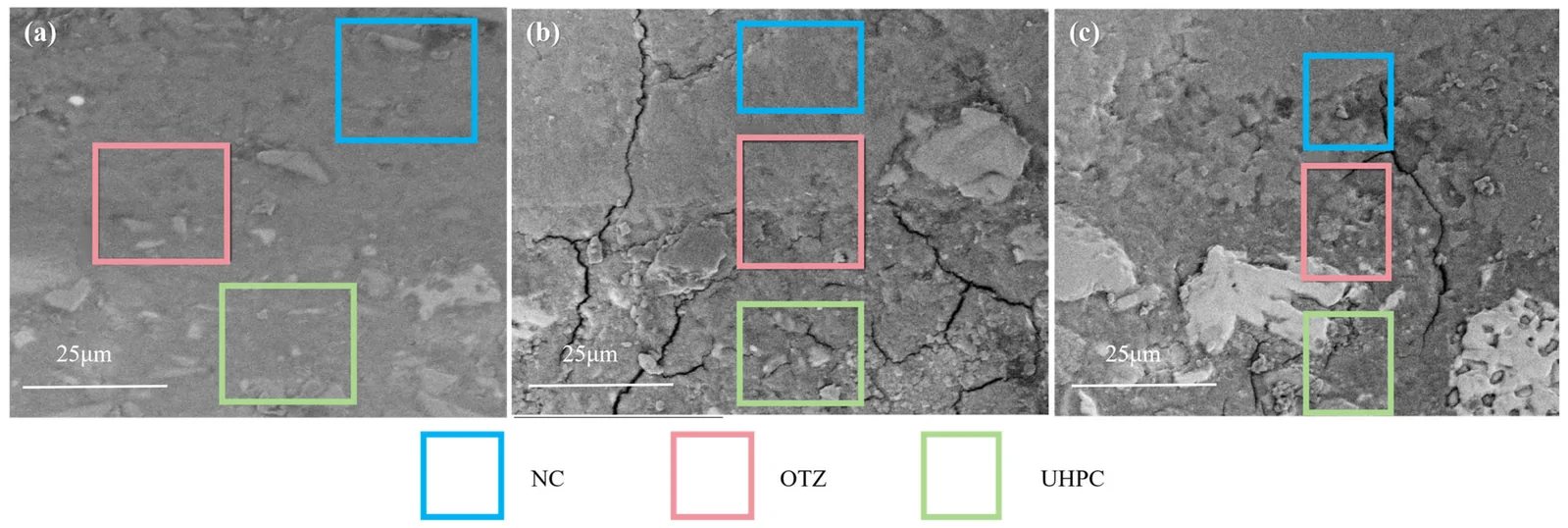
EDS analysis regions within the NC–UHPC near-interface composite region under the different exposure regimes: (**a**) REF, (**b**) C28, and (**c**) C28-SW60.

**Figure 8:**
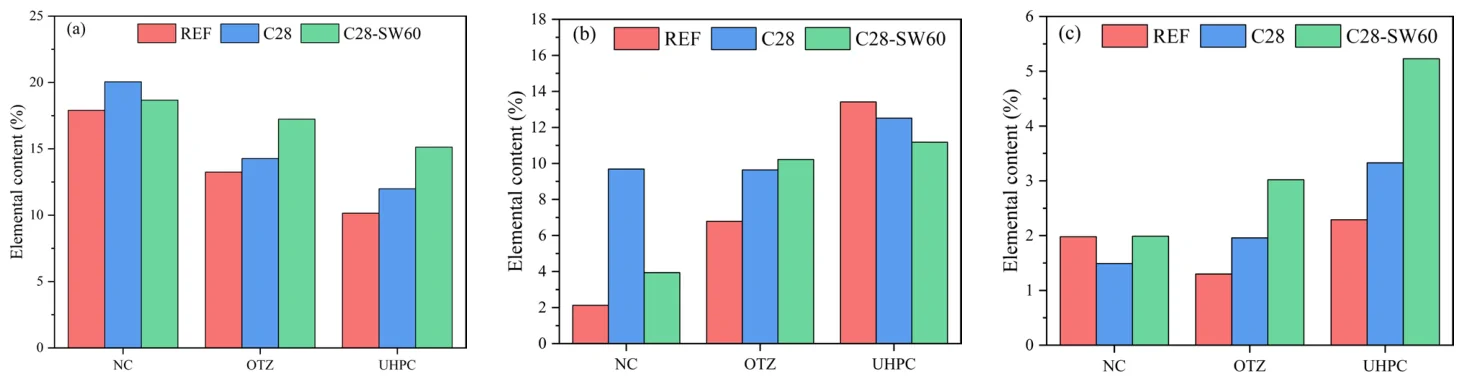

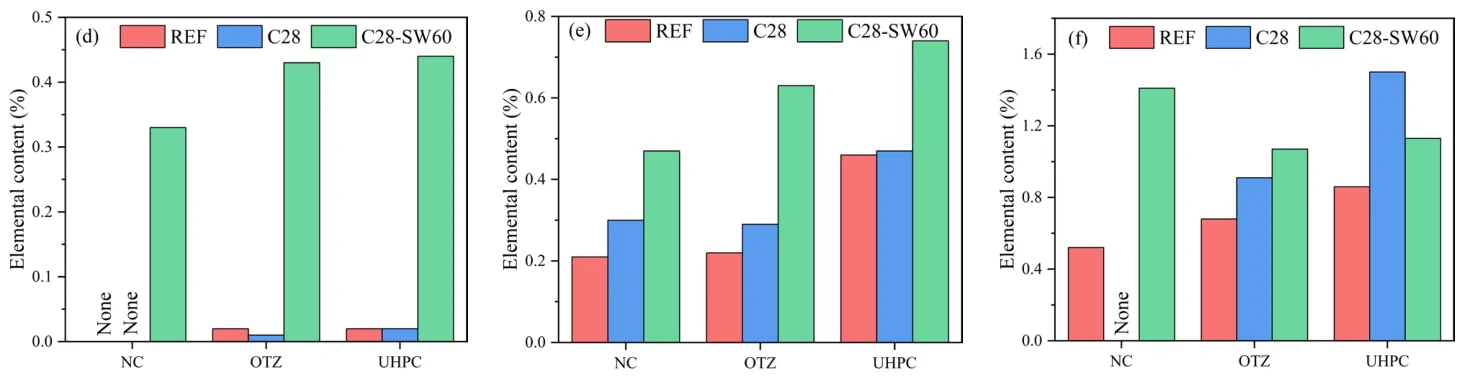
Elemental contents in the NC side, OTZ, and UHPC side under the different exposure regimes: (**a**) Ca, (**b**) Si, (**c**) Al, (**d**) Cl, (**e**) S, and (**f**) Mg.

**Figure 9:**
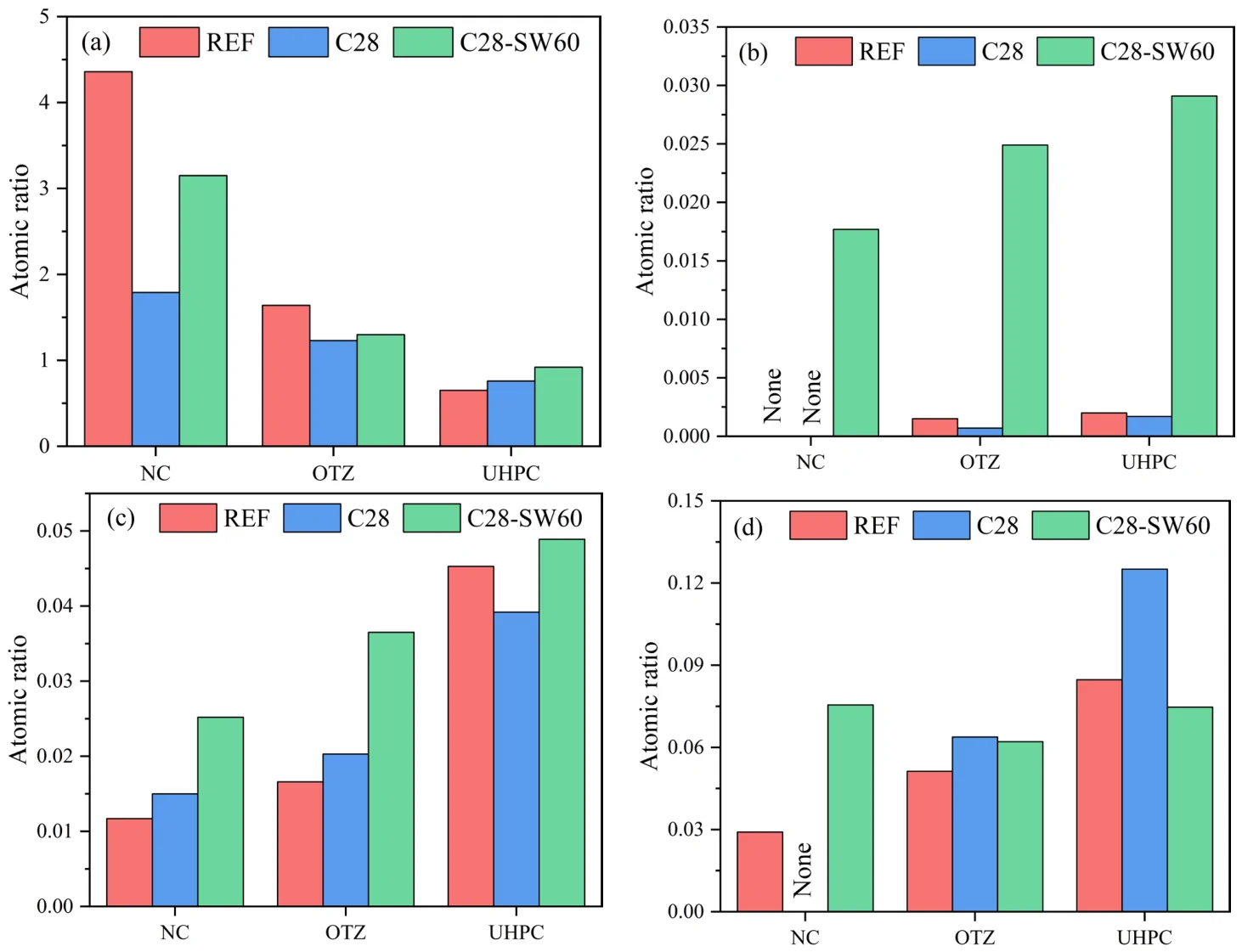
Characteristic atomic ratios in the NC side, OTZ, and UHPC side under the different exposure regimes: (**a**) Ca/(Si + Al), (**b**) Cl/Ca, (**c**) S/Ca, and (**d**) Mg/Ca.

EDS analyses were conducted on selected areas of the NC side, the OTZ, and the UHPC side to compare the elemental compositions and spatial distributions under the different exposure regimes. Figure 7 shows the analyzed regions, while Figure 8 and Figure 9 present the elemental contents and characteristic atomic ratios, respectively. Because the EDS interaction volume may encompass the hydrated matrix, residual unhydrated particles, aggregates, and mineral admixtures, the measured values represent the mixed composition of each selected area and cannot be assigned directly to a single phase.

REF exhibited a pronounced Ca–Si compositional gradient from the NC side through the OTZ to the UHPC side. On the NC side, the Ca, Si, and Al contents were 17.90, 2.13, and 1.98 at.%, respectively, yielding a Ca/(Si + Al) ratio of 4.36 and indicating a strongly Ca-rich composition. In the OTZ, the Ca content decreased to 13.25 at.%, whereas the Si content increased to 6.79 at.% and Ca/(Si + Al) decreased to 1.64. The intermediate composition of the OTZ relative to the two parent materials confirms its chemically transitional character, consistent with previous observations [55]. On the UHPC side, the Ca content decreased further to 10.15 at.%, while the Si content increased to 13.42 at.% and Ca/(Si + Al) declined to 0.65, indicating a distinctly Si-rich composition. This feature likely reflects the high silica-fume content and other siliceous or aluminosilicate constituents in UHPC, the presence of hydration gels with relatively low Ca/Si ratios, and residual siliceous particles [56]. The Cl contents in the three REF regions were only 0.00–0.02 at.%, indicating negligible chloride accumulation before external exposure. By contrast, measurable background concentrations of S and Mg were already present, ranging from 0.21 to 0.46 at.% and from 0.52 to 0.86 at.%, respectively. These background signals originated from the raw materials and their hydration products, particularly the ground granulated blast-furnace slag incorporated into both the NC and UHPC, which contained relatively high MgO and a smaller amount of SO_3_.

After 28 d of accelerated carbonation, the local Ca–Si–Al compositions changed in a region-dependent manner. In the OTZ, Ca/(Si + Al) decreased by approximately 25%, from 1.64 in REF to 1.23 in C28, indicating a reduced relative contribution from Ca-bearing constituents compared with the Si–Al-bearing matrix. By contrast, the ratio on the UHPC side changed only slightly, from 0.65 to 0.76, and remained comparatively low. These results show that carbonation did not produce a uniform decrease in Ca/(Si+Al) throughout the near-interface composite region. When considered together with the attenuation of the crystalline CH reflections and the enhancement of the calcite-related signals in the XRD and TG–DTG results, the EDS data suggest that carbonation altered the relative contributions of Ca-bearing and Si–Al-bearing constituents through CH consumption, CaCO_3_ formation, and the decalcification and restructuring of C–(A)–S–H [44]. However, these changes did not correspond to uniform Ca enrichment or depletion across all three regions. The Cl contents on the NC side, in the OTZ, and on the UHPC side remained at 0.00, 0.01, and 0.02 at.%, respectively, and the corresponding Cl/Ca ratios were negligible, confirming that carbonation alone introduced no detectable external chloride.

The clearest elemental difference in C28-SW60 was the higher Cl content in all three sampled regions. The Cl contents on the NC side, in the OTZ, and on the UHPC side were 0.33, 0.43, and 0.44 at.%, respectively, compared with 0.00–0.02 at.% in REF and C28. The corresponding Cl/Ca ratios in the OTZ and on the UHPC side increased from 0.0015 and 0.0020 in REF to 0.0249 and 0.0291 in C28-SW60. These local EDS measurements indicate chloride ingress across the sampled near-interface region, but they do not quantify bulk chloride uptake or distinguish the chemical state of chloride. The S content increased from 0.21–0.46 at.% in REF to 0.47–0.74 at.% in C28-SW60, while the Mg content increased from 0.52–0.86 to 1.07–1.41 at.%. Because S and Mg were already present in REF and their atomic ratios were affected by local variations in Ca content, these changes are interpreted as enrichment and spatial redistribution superimposed on the initial material background.

Seawater-derived Cl^−^, SO_4_^2−^, and Mg^2+^ can participate in ion transport, chloride binding and release, leaching, and hydrate transformation [57]. Local Cl enrichment does not require the concurrent formation of abundant sulfate-bearing products because EDS detects elements rather than their chemical states. The measured Cl, S, and Mg may have included dissolved or adsorbed species, residual soluble salts, or poorly crystalline and spatially localized reaction products. Consistent with the XRD and TG–DTG results, Friedel’s salt, gypsum, ettringite, brucite, and M–S–H were not detected as dominant crystalline or thermally resolvable products; however, their complete absence cannot be established from the present measurements. Raman spectroscopy could provide additional information on localized reaction products, while chloride titration after acid or water extraction could quantify bulk acid-soluble or water-soluble chloride contents beyond the localized EDS measurements. These complementary analyses were not included in the present study. The available evidence therefore supports elemental ingress and redistribution without showing that crystalline chloride-, sulfate-, or magnesium-bearing reaction products had become dominant.

The BSE observations further showed that the localized deposit-like features, granular accumulations, and connected defects in C28-SW60 were concentrated near cracks, particle boundaries, and discontinuous matrix regions. These features may have facilitated ion transport and local elemental enrichment, although static BSE images alone cannot establish the direction or sequence of transport. The detection of Cl, S, and Mg on the NC side, in the OTZ, and on the UHPC side nevertheless confirms that seawater immersion affected the pre-carbonated near-interface region as an integrated composite zone. Collectively, the XRD, TG–DTG, BSE, and EDS results indicate that sequential pre-carbonation and seawater immersion produced a coupled response involving carbonate enrichment, multi-ion ingress, localized elemental redistribution, and increased morphological heterogeneity.

### 3.3. Pore Structure Evolution

**Figure 10:**
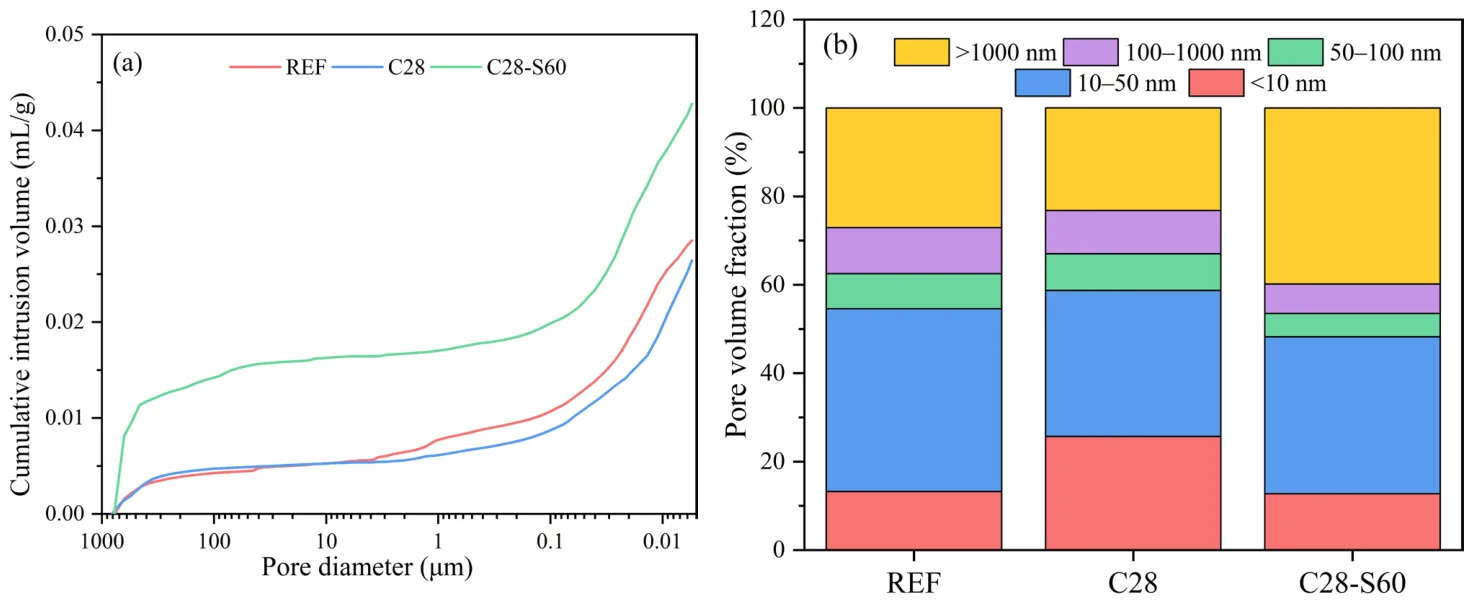
MIP results of the NC-UHPC near-interface composite region: (**a**) cumulative intrusion curves; (**b**) pore-size fraction.

Figure 10 presents the MIP results for the NC–UHPC near-interface composite region, and Table 3 lists the measured pore-structure parameters. REF had a total mercury-intrusion volume of 0.029 mL/g and an MIP-accessible porosity of 6.70%. The corresponding C28 values were 0.026 mL/g and 6.34%, which were 10.3% and 5.3% below the REF values, respectively. The volume-based median pore-entry diameter decreased from 36.54 to 27.49 nm, the average pore-entry diameter decreased from 23.62 to 17.80 nm, and the most probable pore-entry diameter shifted from 13.73 nm to at or below the lower measurable limit (≤5.48 nm). Thus, the C28 specimen combined a modest decrease in mercury-accessible pore volume with a pronounced shift toward finer pore entries. Similar pore refinement with only a limited decrease in total porosity has been reported for carbonated cementitious materials [58,59]. The pore-entry-size fractions support this interpretation. From REF to C28, the <10 nm fraction increased from 13.24% to 25.68%, whereas the 10–50 nm fraction decreased from 41.32% to 33.03%. The 100–1000 nm and >1000 nm fractions also decreased, from 10.40% and 27.07% to 9.76% and 23.21%, respectively. This redistribution is consistent with carbonate formation and the transformation of Ca-bearing hydrates, which can narrow or block accessible capillary-pore entries. When interpreted together with the enhanced calcite-related signals detected by XRD and TG–DTG, this refinement may be attributed primarily to CaCO_3_ formation and the transformation and restructuring of Ca-bearing hydrates, including CH and C–(A)–S–H [44,58,60]. The carbonation of C–(A)–S–H involves decalcification and the formation of CaCO_3_ and Si-rich gel, thereby modifying the pore structure of the binding matrix [44,60]. The BSE images nevertheless showed local particle boundary cracks and interfacial discontinuities. Finer MIP pore entries therefore coexisted with localized defects, indicating heterogeneous refinement rather than uniform densification [61].

After artificial seawater immersion, the measured MIP parameters shifted toward a coarser mercury-accessible pore structure. C28-SW60 had a total mercury intrusion volume of 0.043 mL/g and an MIP-accessible porosity of 9.56%, compared with 0.026 mL/g and 6.34% for C28. These values were 65.4% and 50.8% above the C28 values, respectively. The volume-based median, average, and most probable pore-entry diameters in C28-SW60 were 58.42, 26.95, and 21.09 nm, respectively. The >1000 nm pore-volume fraction increased from 23.21% in C28 to 39.82% in C28-SW60, whereas the <10 nm fraction decreased from 25.68% to 12.74%. Thus, C28-SW60 did not retain the pore-entry refinement observed in C28. Previous studies have likewise shown that seawater exposure can produce stage-dependent microstructural responses: continued hydration and localized precipitation may initially refine pores, whereas later chemical alteration, volumetric deformation, and microcracking may coarsen them [23,37].

The shift toward coarser mercury-accessible pore entries in C28-SW60 was accompanied by more pronounced particle-boundary discontinuities, slit-like defects, and locally connected crack-like features in the BSE images. A similar association between increased cube-scale porosity measured by micro-CT and a less dense microscopic structure has been reported for sulfate-exposed concrete [62]. Although micro-CT resolves the geometry and spatial distribution of larger voids, whereas MIP characterizes mercury-accessible pore entries, the corresponding trends observed across these scales indicate that the coarser MIP response of C28-SW60 was associated with the development of local defects rather than with a change in total porosity alone. These structural changes coincided with substantial Cl ingress and further S and Mg redistribution, while XRD and TG–DTG showed carbonate-related phase evolution without dominant crystalline salt-attack products. Overall, C28-SW60 showed an adverse microstructural shift relative to C28, marked by carbonate enrichment, ion ingress, coarser mercury-accessible pore entries, and locally connected crack-like features.

## 4. Discussion

### Microstructural Evolution Mechanism

**Figure 11:**
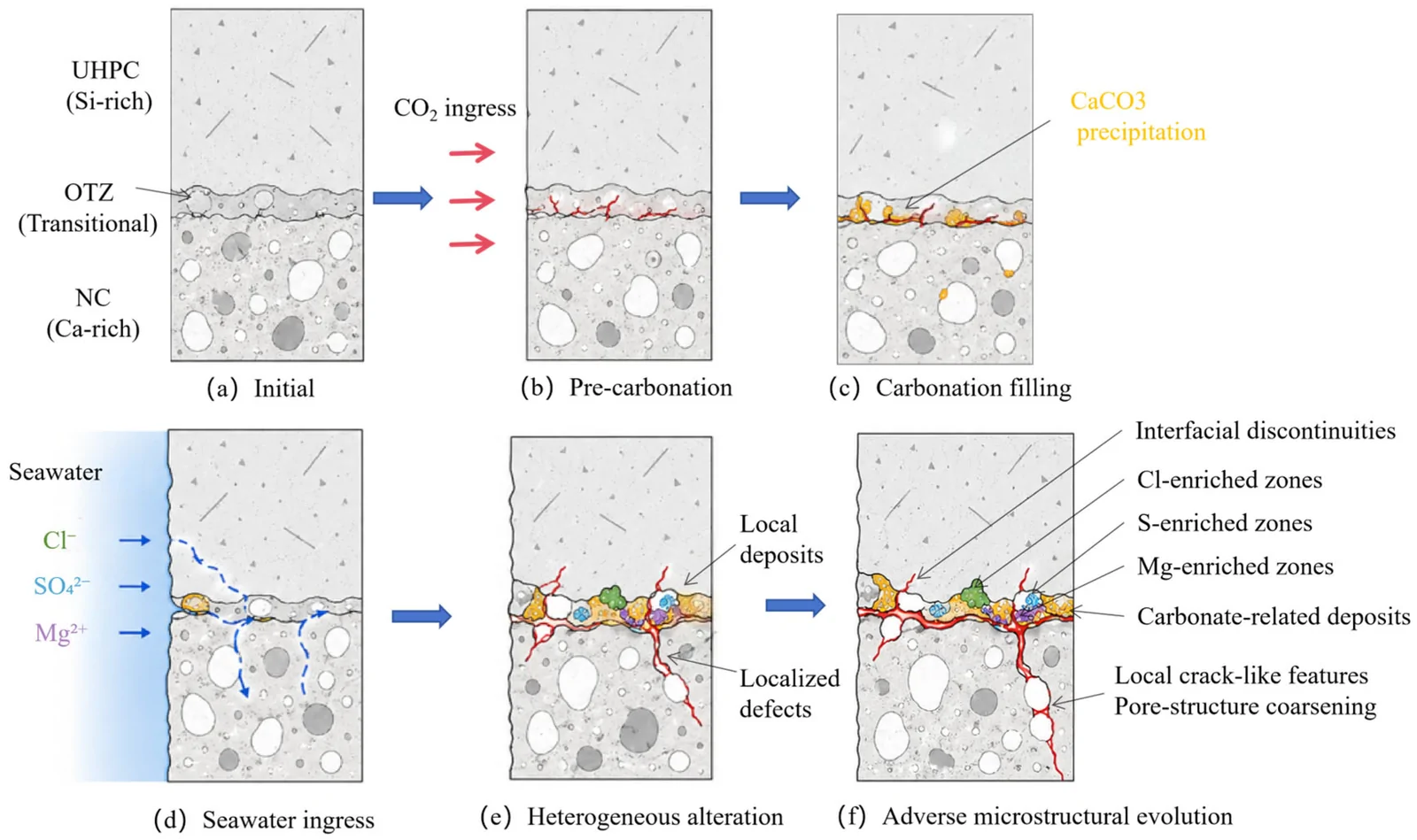
Proposed mechanism governing the microstructural evolution of the NC–UHPC near-interface composite region under sequential pre-carbonation and seawater immersion.

The combined results show that the initial heterogeneity of the NC–UHPC near-interface region governed its sequential response to pre-carbonation and seawater immersion. Figure 11 summarizes the proposed mechanism, which links phase transformation and multi-ion redistribution to changes in morphology and mercury-accessible pore entries. The NC side, the OTZ, and the UHPC side exhibit Ca-rich, chemically transitional, and Si-rich characteristics, respectively, and differ substantially in hydrate assemblage, pore connectivity, and initial defect distribution. Consequently, CO_2_ and seawater-derived ions do not penetrate the near-interface region through a spatially uniform reaction front. Instead, their transport is preferentially associated with the OTZ, particle boundaries, connected pores, and localized interfacial defects [21,63]. This initial chemical and structural heterogeneity governs the subsequent transport and reaction processes.

During pre-carbonation, CO_2_ enters the near-interface region through preferential transport pathways and reacts with CH and other Ca-bearing hydrates, including C–(A)–S–H. CH consumption and C–(A)–S–H decalcification promote the formation of CaCO_3_ and Si-rich gel, thereby reorganizing both the solid-phase assemblage and the pore structure [58,60,64]. Carbonate precipitation can fill or constrict part of the capillary-pore network and shift the mercury-accessible pore population toward finer pore-entry sizes. However, spatial variations in Ca availability, reaction extent, and initial defect distribution prevent the formation of a continuous and uniformly densified layer. The pre-carbonated near-interface region therefore exhibits a heterogeneous microstructure in which overall pore refinement coexists with local particle-boundary cracking and interfacial discontinuities. Pre-carbonation also alters pore-solution alkalinity and the chemical stability of hydrates such as AFm and C–(A)–S–H and may reduce chloride-binding capacity and destabilize chloride-bearing aluminate phases [65,66].

During subsequent seawater immersion, Cl^−^, SO_4_^2−^, and Mg^2+^ enter the pre-carbonated near-interface region through the remaining connected pores and localized defects. The pronounced increase in Cl provides direct evidence of external chloride ingress, whereas the increases in S and Mg indicate further enrichment and redistribution relative to their initial material backgrounds. Because transport pathways and local chemical reactivity vary spatially, these elements do not accumulate uniformly but instead produce pronounced compositional heterogeneity across the NC side, the OTZ, and the UHPC side, particularly near particle boundaries and discontinuous matrix regions. Seawater-derived ions can further promote the leaching of Ca-bearing constituents, decalcification of binding phases, and localized dissolution–transport–reprecipitation, thereby altering the solid assemblage and restructuring the pore–crack network [30,57]. These processes can account for the coexistence of enhanced carbonate-related signals, localized deposit-like features, particle-boundary defects, and connected cracks.

Connected pores, particle boundaries, and local discontinuities provided preferential paths for Cl^−^, SO_4_^2−^, and Mg^2+^ to enter the near-interface region [21,57]. Once inside, these ions could adsorb on or enter existing hydrates, remain as pore-solution species or residual salts after drying, or participate in localized dissolution–reprecipitation [30]. These forms can retain the elements without producing abundant, well-crystallized reaction products. EDS detected the elements locally but could not distinguish among their host environments. Conventional XRD may not resolve poorly crystalline, amorphous, low-abundance, or spatially restricted products, and bulk powder sampling can further dilute products confined to local reaction zones [67]. The absence of distinct reflections therefore indicates that Friedel’s salt, gypsum, ettringite, brucite, and M–S–H did not dominate the crystalline phase assemblage; it does not establish that minor or poorly crystalline forms were absent. These differences in observation scale explain why local elemental enrichment coexisted with a bulk phase assemblage that lacked a dominant crystalline salt-attack product. They also support the proposed mechanism of heterogeneous ion transport and retention in the pre-carbonated near-interface region. Taken together, the phase, thermal, morphological, elemental, and pore structure data show that localized precipitation did not preserve the MIP pore refinement observed after pre-carbonation. After multi-ion exposure, the near-interface region showed greater chemical and structural heterogeneity, coarser mercury-accessible pore entries, and local crack-like discontinuities. The proposed mechanism comprises three stages. Initial chemical and structural heterogeneity defines preferential transport paths. Pre-carbonation transforms Ca-bearing hydrates and refines mercury-accessible pore entries. Subsequent ion ingress promotes localized leaching, decalcification, and dissolution–reprecipitation. These processes can explain the observed pore coarsening and local crack-like features.

Previous studies have shown that carbonation alters chloride transport in concrete [26,27,28,29]. Other studies have shown that multi-ion seawater exposure changes hydrate assemblages in cementitious materials [30,31,32,33,34,35,36]. The present study connects these processes within the chemically and structurally heterogeneous NC–OTZ–UHPC near-interface region. The combined evidence indicates that the chemical gradient and defect structure present before seawater exposure conditioned the subsequent redistribution of seawater-derived ions. Carbonate enrichment coexisted with coarser mercury-accessible pore entries and more pronounced crack-like features, although crystalline salt-attack products were not detected as dominant. This sequence-dependent response distinguishes carbonate accumulation from sustained near-interface refinement and identifies initial heterogeneity and exposure history as the organizing factors in the observed microstructural evolution.

The proposed mechanism is bounded by the experimental design. It describes one prescribed sequence—pre-carbonation followed by accelerated artificial seawater immersion—and does not establish the response of an uncarbonated interface to seawater or a time equivalence with natural marine service. Moreover, the measurements relate phase transformation and elemental redistribution to morphological changes and shifts in mercury-accessible pore entries; they do not quantify corresponding changes in interfacial bond strength, transport performance, or service life. Within these boundaries, the study provides a mechanism-based account of how a pre-carbonated NC–UHPC near-interface region evolves during subsequent multi-ion exposure.

## 5. Conclusions

In this study, the XRD, TG–DTG, BSE–EDS, and MIP were used to elucidate the microstructural evolution of the NC–UHPC near-interface composite region under sequential pre-carbonation and seawater immersion. The main conclusions are as follows:

(1) Pre-carbonation promoted CH consumption and carbonate formation, whereas subsequent seawater immersion further enhanced the calcite diffraction and high-temperature carbonate decomposition signals. Under the present exposure conditions, Friedel’s salt, gypsum, ettringite, brucite, and M–S–H were not detected as dominant crystalline products.
(2) REF exhibited an initial chemical gradient from the Ca-rich NC side, through the chemically transitional OTZ, to the Si-rich UHPC side. After seawater immersion, Cl accumulated across all three sampled regions, while S and Mg underwent further enrichment and redistribution relative to their initial material backgrounds. This spatial response indicates that subsequent multi-ion exposure interacted with the entire near-interface composite region rather than with a single constituent material.
(3) MIP indicated that pre-carbonation was associated with finer mercury-accessible pore entries and a modest decrease in total intrusion volume. After seawater immersion, the total intrusion volume increased from 0.026 to 0.043 mL/g, the volume-based median pore-entry diameter increased from 27.49 to 58.42 nm, and the >1000 nm fraction increased from 23.21% to 39.82%. Considered with the BSE observations, these changes indicate a reversal of the pore refinement trend and a shift toward coarser transport-relevant defects.
(4) Initial chemical and structural heterogeneity defines preferential transport paths within the NC–UHPC near-interface region. Pre-carbonation transforms Ca-bearing hydrates and promotes carbonate precipitation, whereas subsequent multi-ion ingress promotes localized leaching, decalcification, dissolution–reprecipitation, and pore–crack restructuring. The resulting early microstructural deterioration state combines carbonate enrichment, elemental redistribution, and coarser mercury-accessible pore entries. Thus, carbonate enrichment or localized pore filling alone does not establish improved interfacial durability; connected defects and ion-transport pathways must also be considered when assessing NC–UHPC repair systems.

## Figures and Tables

**Table 1:** Mix proportions of the NC substrate and the UHPC overlay (kg/m^3^) [37].

	Cement	Water	Coarse Aggregate	Sand	Steel Fiber	Water-Reducing Admixture (No. 1)	Water-Reducing Admixture (No. 2)	Ground Granulated Blast-Furnace Slag	Limestone Powder	Silica Fume
NC	192	163	940	868	/	10.9	/	134	92	/
UHPC	833	210	/	1042	153	/	31	200	/	130

**Table 2:** Chemical compositions of the cementitious materials [37].

Materials		Cement	Limestone Powder	Silica Fume	Ground Granulated Blast-Furnace Slag
Chemical composition (%)	CaO	63.69	51.94	1.51	34.99
	MgO	2.71	1.17	4.07	10.57
	SO_3_	0.24	0.39	0.26	0.49
	SiO_2_	23.14	4.69	80.96	26.56
	Al_2_O_3_	4.37	1.01	1.89	16.36
	Fe_2_O_3_	4.03	0.47	2.4	0.52
	f-CaO	0.46	/	/	/
	K_2_O + Na_2_O	0.43	0.10	0.27	0.13

**Table 3:** MIP parameters of the NC-UHPC near-interface composite region.

Group	Total Intrusion Volume (mL/g)	Porosity (%)	Most Probable Pore Diameter (nm)	Median Pore Diameter by Volume (nm)	Average Pore Diameter (nm)
REF	0.029	6.70	13.73	36.54	23.62
C28	0.026	6.34	≤5.48	27.49	17.80
C28-SW60	0.043	9.56	21.09	58.42	26.95

## Data Availability

The original contributions presented in this study are included in the article. Further inquiries can be directed to the corresponding author.

## Nomenclature

The following nomenclature is used in this manuscript:

UHPC	Ultra-high-performance concrete
NC	Normal concrete
OTZ	Overlay transition zone
REF	Unexposed reference condition
C28	Accelerated carbonation for 28 days
C28-SW60	Accelerated carbonation for 28 days followed by artificial seawater immersion for 60 days
XRD	X-ray diffraction
TG	Thermogravimetry
DTG	Derivative thermogravimetry
BSE–EDS	Backscattered electron imaging coupled with energy-dispersive X-ray spectroscopy
MIP	Mercury intrusion porosimetry
CH	Portlandite
C–(A)–S–H	Calcium (alumino)silicate hydrate
AFt	Ettringite-type calcium aluminate hydrate
AFm	Monosulfate-type calcium aluminate hydrate
M–S–H	Magnesium silicate hydrate
